# scTyper: a comprehensive pipeline for the cell typing analysis of single-cell RNA-seq data

**DOI:** 10.1186/s12859-020-03700-5

**Published:** 2020-08-04

**Authors:** Ji-Hye Choi, Hye In Kim, Hyun Goo Woo

**Affiliations:** 1 Department of Physiology, Ajou University School of Medicine, 164 Worldcup-ro, Yeongtong-gu, Suwon, 16499 Republic of Korea; 2 Department of Biomedical Science, Graduate School, Ajou University, Suwon, Republic of Korea

**Keywords:** Single-cell RNA sequencing, Cell typing, Cell type marker database

## Abstract

**Background:**

Recent advances in single-cell RNA sequencing (scRNA-seq) technology have enabled the identification of individual cell types, such as epithelial cells, immune cells, and fibroblasts, in tissue samples containing complex cell populations. Cell typing is one of the key challenges in scRNA-seq data analysis that is usually achieved by estimating the expression of cell marker genes. However, there is no standard practice for cell typing, often resulting in variable and inaccurate outcomes.

**Results:**

We have developed a comprehensive and user-friendly R-based scRNA-seq analysis and cell typing package, scTyper. scTyper also provides a database of cell type markers, scTyper.db, which contains 213 cell marker sets collected from literature. These marker sets include but are not limited to markers for malignant cells, cancer-associated fibroblasts, and tumor-infiltrating T cells. Additionally, scTyper provides three customized methods for estimating cell-type marker expression, including nearest template prediction (NTP), gene set enrichment analysis (GSEA), and average expression values. DNA copy number inference method (inferCNV) has been implemented with an improved modification that can be used for malignant cell typing. The package also supports the data preprocessing pipelines by Cell Ranger from 10X Genomics and the Seurat package. A summary reporting system is also implemented, which may facilitate users to perform reproducible analyses.

**Conclusions:**

scTyper provides a comprehensive and user-friendly analysis pipeline for cell typing of scRNA-seq data with a curated cell marker database, scTyper.db.

## Background

Single-cell RNA sequencing (scRNA-seq) technology has enabled researchers to profile transcriptomes at single-cell level [1, 2]. However, there are a number of challenges in the analysis of scRNA-seq data and its outcomes; one of the key challenges is the identification of cell types from the transcriptome data. Currently, various cell typing methods have been introduced using different workflows and data types [2–6]. Cell typing by estimation of the expression level of cell marker genes is generally used by researchers for convenience. With time, enriched resources for cell type markers that have been generated from different sources, including single cell sequencing and experimental studies, are becoming available [7, 8]. Thus, cell typing using these inconsistent markers has become more time-consuming and an error-prone process. Thus far, there is no standard practice for cell typing and use of different cell markers and cell typing algorithms often results in inconsistent cell type assignment.

To overcome this issue, a collection of versatile cell markers from previous studies is needed for cell typing. In fact, there is a comprehensive cell marker database, CellMarker, which provides manually curated cell markers and their information [9]. However, this database does not include the recent studies, especially on tumor tissues, even though many tumor-associated cells have been characterized recently [10–12].

In this study, we developed scTyper, an R package that provides a cell marker database, scTyper.db, as well as a flexible pipeline for cell typing analysis of scRNA-seq data with three different methods. Users can customize the cell typing pipeline and easily use the pre-collected cell marker databases.

### Implementation

scTyper is an R package that can be executed by a single command. Experienced users can customize the pipeline stepwise by manipulating the parameters. Besides the cell typing process, scTyper also supports pipelines for quality control and sequence alignment, which are performed by FASTQC (https://www.bioinformatics.babraham.ac.uk/projects/fastqc) and Cell Ranger [13], respectively. Data normalization, clustering, and visualization processes are also supported by the wrapper functions for ‘Seurat’ R package [14].

## Results

### Overall workflow of scTyper

**Fig. 1:**
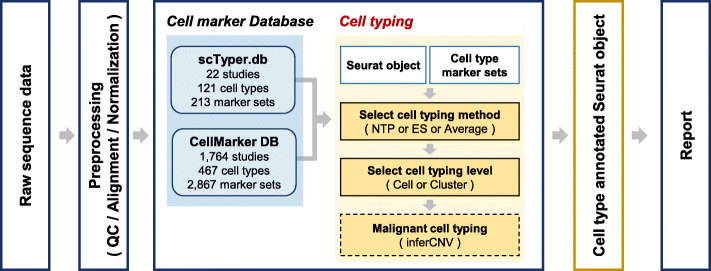
Overall workflow of scTyper. A schematic diagram of the workflow of scTyper for scRNA seq-based cell typing

scTyper provides an automated and customizable pipeline for the cell typing of scRNA-seq data (Fig. 1). For user convenience, the package has been supported with raw data preprocessing pipelines by wrapper functions for FASTQC and Cell Ranger from 10X Genomics; the preprocessing includes quality control, sequence alignment and quantification of raw sequencing data. These processes can be executed by a single command. Data processing steps for log transformation, normalization, and clustering are performed by the wrapper functions for Seurat, generating a Seurat object that is used as an input file in the subsequent processes.

After data processing, cell typing can be performed using the pre-pooled cell marker database, scTyper.db, and a previously reported cell marker database, CellMarker [9]. Users can choose the cell markers of interest from these databases and apply them to subsequent cell typing. The expression of the cell marker sets can be estimated by three different methods, nearest template prediction (NTP) [15], pre-ranked gene set enrichment analysis (GSEA) [16], and average gene expression values. For malignant cell typing, users can utilize the inferred DNA copy numbers using the inferCNV R package with modifications [17].

Overall, scTyper is comprised of the modularized processes of “QC”, “Cell Ranger”, “Seurat processing”, “cell typing”, and “malignant cell typing”. These processes can be customized by manipulating the parameters for each process. If users want to perform only the cell typing process and a preprocessed input file with Seurat object is already prepared, the processing steps of “QC”, “Cell Ranger” and “Seurat processing” can be skipped by setting the parameters “qc”, “run.cellranger” and “norm.seurat” to “FALSE”. The processes and their parameters implemented in scTyper are summarized in **Supplementary Table** **1** (more details can be found in the package manual).

Finally, the results and the executed processes are automatically documented as a report. The report summarizes the processing steps, cell typing and clustering results, and visualizes the results with plots.

### scTyper.db, a manually curated cell marker database

**Fig. 2:**
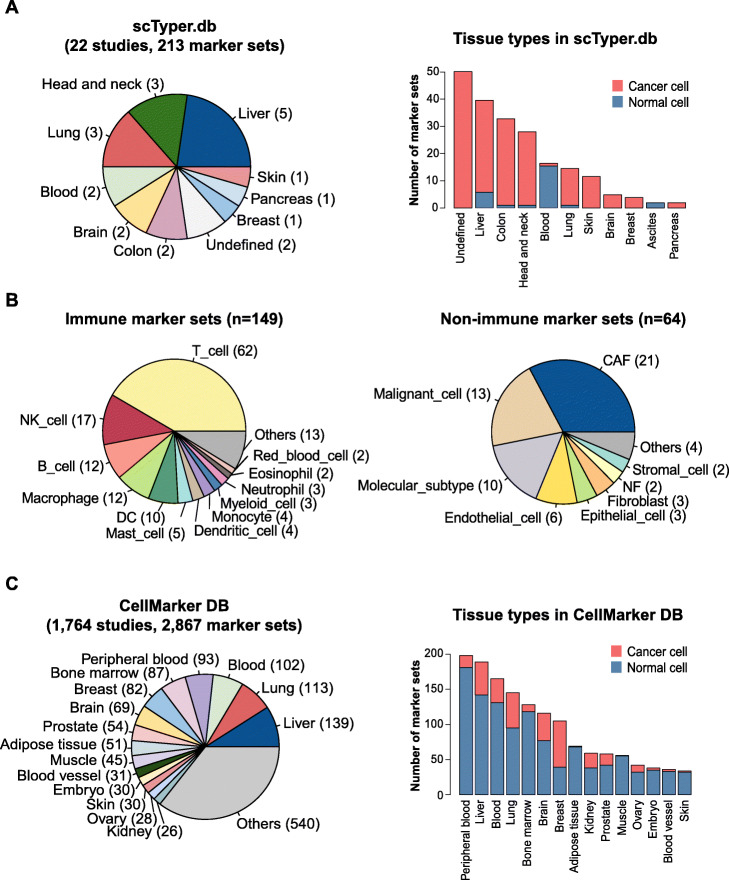
The manually curated cell marker databases in scTyper. **a.** A pie plot shows the proportion of the tissue types in scTyper.db (*left*). A bar plot indicates the number of gene sets across tissue types in scTyper.db (*right*). Malignant (*red*) and non-malignant (*blue*) cell types are indicated with different colors. **b.** Pie plots show the proportion of the immune (*left*) and non-immune cell (*right*) markers implemented in scTyper.db. **c.** A pie plot shows the proportion of the tissue types in CellMarker (*left*). A bar plot indicates the number of gene sets across tissue types in CellMarker (*right*). Malignant (*red*) and non-malignant (*blue*) cell types are indicated with different colors

scTyper.db is pre-installed in the package that is comprised of manually curated 213 cell marker gene sets and the 121 cell types collected from 22 studies (**Supplementary Table** **2**). We collected the cell markers for cancer-associated fibroblasts (*n* = 21), tumor-infiltrated lymphocyte (*n* = 33), tumor-associated macrophage (*n* = 4), and malignant cells from different tissue types (*n* = 13) (Fig. 2a-b and **Supplementary Table** **2****)**. Immune repertoires of 149 immune cell markers were also included in the database. For example, there were 62 T cell marker sets with different cell transition states such as CD4+, CD8+, regulatory T, and exhausted T cells.

We have used a unified nomenclature to label the marker gene sets in the database. For example, a cell marker label “Puram.2017.HNSCC.TME” was designated by concatenating the first author name of the publication (Puram), publication year (2017), tissue type/cancer type (HNSCC), and category of cell composition (TME, tumor microenvironment). Using this nomenclature, users can easily search the cell markers of interest. Detailed information about the cell markers such as data source, PubMed ID, species name, tissue type, study detail, etc. was also provided in the “extdata” directory. In addition to scTyper.db, we also implemented the previous, CellMarker database, which comprised 2867 cell type marker sets and 467 cell types from 1764 studies (Fig. 2c and **Supplementary Table** **3**).

### Cell marker expression estimation and cell typing

In the current version of scTyper, three different methods are implemented to estimate the expression of cell marker sets, including NTP, pre-ranked GSEA, and average expression values (Fig. 1). NTP is a class prediction method to estimate the proximity to the cell type templates by using a list of gene sets and calculating its distance to the test data [18]. Enrichment score (ES) is calculated by the pre-ranked GSEA method (https://www.gsea-msigdb.org/gsea/index.jsp). Users can choose the level for cell typing from the options, “cell-level” or “cluster-level” by setting the value of the parameter “level” to “cell” or “cluster”, respectively.

For malignant cell typing, inferred DNA copy numbers are estimated by the inferCNV R package [17] with an improved modification. The group of genes with same function can be located within their proximity on a chromosome, resulting in the construction of a gene cluster. These gene clusters can have similar expression levels and thus can be falsely inferred to have regional DNA copy number alteration. Therefore, we have added a gene filtering step in the inferCNV process to remove gene clusters from the inferCNV analysis.

**Fig. 3:**
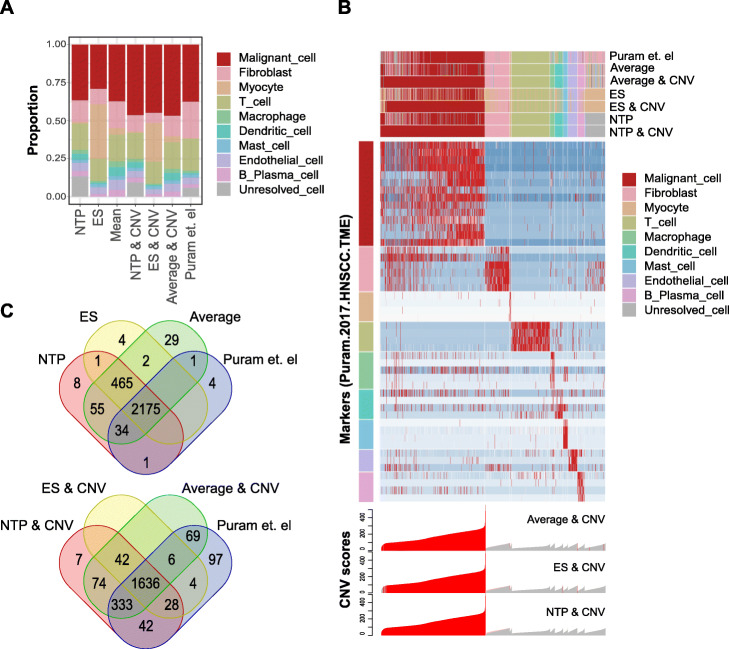
Cell typing with different cell typing options in scTyper. **a.** A bar plot shows the proportion of cell type assignments by using the six methods according to the use of different cell typing methods (i.e., NTP, ES, and average values) and the inferCNV-based malignant cell typing. The results are also compared with that of the original study (Puram et al.). **b.** A heatmap shows the cell typing result with the gene expression levels of the 9 cell types of ‘Puram.2017.HNSCC.TME’. For each method, the assigned cell types are indicated by color bars (*top*). The inferred CNV values for each cell are shown (*bottom*). The cells with the CNV values greater than 90 percentile of the CNV values of immune cells were considered to have DNA copy number aberration and designated as malignant cells (red color bars). The malignant cells were assigned by cell typing methods (predicted as ‘Epithelial cell’) or CNV values (CNV values for reference cells > Q90, i.e., 86.44). **c.** Venn diagrams show the overlapped counts of the malignant cells assigned by the three cell typing methods without (*top*) or with (*bottom*) inferCNV analysis. The cell type assignment from the original study (Puram et al.) is also compared

Next, we benchmarked the performance of different cell typing methods implemented in scTyper using a test data set (GSE103322, 5902 cells from head and neck squamous cancer) [18]. Cell typing was performed with 6 different parameters using all the 3 cell typing methods for comparison with or without the application of inferCNV. The cell markers of Puram.2017.HNSCC.TME were used. As expected, we observed that the cell types were assigned differently based on the methods applied (Fig. 3a). For instance, applying the inferCNV method could identify 529 additional malignant cells that were assigned to be non-malignant cells in the original Puram study (Fig. 3b, c). During inferCNV analysis, 5 gene clusters (including 180 genes) were filtered out; these were identified by performing gene set enrichment analysis of genes residing in the neighboring chromosomal regions (1 Mb) (*P* < 0.05). These results show that the combined analysis of the cell marker expression and CNV inference is greatly helpful in appropriately interpreting the cell typing results.

In the performance test, cell typing of the test data (5902 cells) with NTP and inferCNV utilized 2.25 h of runtime under the computing environment of a single CPU core (Intel Xeon, 2.40 GHz) and 500 M RAM (**Supplementary Fig.** **1**). Most of the runtime was used by the inferCNV (1.43 h) and NTP (0.32 h) processes. We also tested a larger test set with 54,239 cells (in-house data), that utilized 20.63 h of runtime. Preprocessing steps for raw data (“QC” and “Cell Ranger”) were not included in the performance test. Parallel computation using multiple CPU cores (up to 20) could enhance the performance, improving the runtime to 0.47 h for 5902 cells and 5.47 h for 54,239 cells.

The scTyper generates an automatic report summary document (**Supplementary Data**); this document summarizes each step of the processes including the parameters used and the results of cell typing and clustering, and visualization plots (heatmaps and UMAP/t-SNE plots). This may help users reproduce their analysis workflows.

## Discussion

In this study, we employed a comprehensive and flexible pipeline for cell typing of scRNA-seq data, by providing manually curated, pre-installed cell marker databases and three different cell typing methods. Customization or update of the cell marker database can be easily accomplished by replacing the ‘sigTyper.db.txt’ file in the “extdata” directory to a newer one. The package allows the users to use and compare different cell typing methods. The modularized design of the pipeline enables users to modify the pipeline at each step, will facilitating the appropriate interpretation of data.

scTyper has some limitations for implementation in the current version. The package does not include the cell typing methods that utilize reference scRNA-seq data instead of the cell markers [4, 5]. Divergent clustering and dimension reduction methods can be applied to the analysis pipeline, but the current version of scTyper only supports the functions provided by the “Seurat” package such as “PCA” or “UMAP/t-SNE”.

## Conclusions

We developed scTyper, a flexible and user-friendly pipeline for cell typing of scRNA-seq data. This package can help users to perform reproducible and comprehensive cell typing.

### Availability and requirements

**Project name**: scTyper.

**Project home page**: https://github.com/omicsCore/scTyper

**Operating system**: Linux dependent.

**Programming language**: R.

**Other requirements:** R 3.5 or higher.

**License**: GPL2.

**Any restrictions to use by non-academics:** None.

## Supplementary information

**Additional file 1: Supplementary Table 1.** Parameters for scTyper.

**Additional file 2: Supplementary Table 2–3.** This file contains the list of cell markers in each of scTyper.db (Table S2) and CellMarker DB (Table S3) and detailed information such as identifier, study name, species, cell type, gene symbol, and PMID.

**Additional file 3: Supplementary Figure. 1.** Runtime of scTyper according to CPU cores up to 20. A plot shows runtimes for cell typing pipeline of scTyper according to the CPU cores (up to 20). The “NTP” cell typing method and inferCNV were applied for the test.

**Additional file 4: Supplementary Data.** An example report summary document of scTyper.

## Data Availability

Source codes and a detailed manual are freely available at https://github.com/omicsCore/scTyper

## Abbreviations

scRNA-seq: Single-cell RNA sequencing
NTP: Nearest template prediction
GSEA: Gene set enrichment analysis
CNV: Copy number variation
ES: Enrichment scores
CAF: Cancer associated fibroblast
TIL: Tumor infiltrated lymphocyte
