# Supplementary material for: scTyper: a comprehensive pipeline for the cell typing analysis of single-cell RNA-seq data
Source: BMC Bioinformatics. 2020 Aug 4;21:342. doi: 10.1186/s12859-020-03700-5 (PMC7430822; doi:10.1186/s12859-020-03700-5)
Supplement: Supplementary file 1 — Additional file 1: Supplementary Table 1. Parameters for scTyper. [file 12859_2020_3700_MOESM1_ESM.docx]

**Supplementary Table**

**Supplementary Table 1. Parameters for scTyper**

| Process | Parameters | Description | Values |
| --- | --- | --- | --- |
| global configuration | wd | Working directory | Character |
|  | output.name | Output directory name | Character |
|  | pheno.fn | Phenotype file path | File path |
|  | qc  run.cellranger  norm.seurat | Indicate whether the process run | Logical (Default = ‘FALSE’) |
|  | cell.typing.method | Cell typing method | ‘NTP’ (Default), ‘ES’, ‘Average’ |
|  | level | Indicate the cell assignment level (cell or cluster) | ‘cell’ (Default), ‘cluster’ |
|  | run.inferCNV | Indicate whether ‘malignant cell typing by inferCNV process run | Logical  (Default = ‘TRUE’) |
|  | project.name | Project name | Character |
|  | mc.cores | Number of cores | Numeric (Default = ‘1’) |
|  | report.mode | Generate report file | Logical (Default = ‘TRUE’) |
| QC | fastqc.path | FastQC program path | File path |
|  | fastq.dir | FastQC output directory | File path |
|  | fq1.idx | Index of the FASTQ file (Read 1) | Character (Default = ‘_R1_001.fastq’) |
|  | fq2.idx | Index of the FASTQ file (Read 2) | Character (Default = ‘_R2_001.fastq’) |
| Cell Ranger | cellranger.path | Cell Ranger program path | File path |
|  | cellranger.ref.dir | Directory of Cell Ranger reference file | File path |
| Seurat processing | percent.min.cells | Cutoff to filter features containing minimum percent of cells | 0.1 |
|  | min.features | Cutoff to filter cells containing minimum number of features | 200 |
|  | percent.mt | Cutoff for filtering cells that have >n percent mitochondrial counts | 10 |
|  | vars.to.regress | Variables to regress out | Default=c(‘nCount_RNA’, ‘percent.mt’) |
|  | dims | A vector of the dimensions to use in construction of the SNN graph. | 1:100 |
|  | resolution | Value of the resolution parameter, use a value above (below) 1.0 if you want to obtain a larger (smaller) number of communities. | 2 |
| Cell typing | seurat.object | Seurat object | Seurat object |
|  | slot | Data type of Seurat object | ‘scale.data’ (Default), ‘count.data’, ‘data’ |
|  | marker | Cell markers to use cell typing | Character or List (Signature names or Study names or User defined gene set list) |
|  | assay | Assay of Seurat object | Character  (Default=’RNA’) |
|  | NTP.g.filter.method | Method to filter genes in NTP | ‘sd’ (Default),’mad’, ‘none’ |
|  | NTP.gene.filter.cutoff | Cutoff to filter genes of in NTP | Numeric (Default = ‘0.3’) |
|  | NTP.distance | NTP distance method | ‘cosine’ (Default), ‘correlation’ |
|  | NTP.norm.method | NTP normalization method | ‘none’ (Default), ‘row.std’ |
| Malignant cell typing (inferCNV) | gene.ref.gtf | Path of GTF file including genomic location for genes | File path |
|  | feature.to.test | Column header name of the meta data in Seurat object (select the cell groups for T.test) | Character  (Default = ‘cell.type’), ‘tissue.type’ |
|  | cells.test_excluded | A value indicates the cells to be excluded in T.test | Character  (Default = ‘Epithelial’) |
|  | cells.test_reference | A value indicates the cells to use as be excluded in T.test | character  (Default = ‘immune’) |
|  | fc.cutoff | Cutoff of fold change | Numeric (Default = ‘0.05’) |
|  | cutoff.gene.cluster | A cutoff P-value for filtering out the gene clusters (calculated from GO analysis) | Numeric (Default = ‘0.05’) |
|  | malignant.cell.type | Cell type to assign malignant cell | Character  (Default = ’Epithelial’) |
