# Supplementary material for: scTyper: a comprehensive pipeline for the cell typing analysis of single-cell RNA-seq data
Source: BMC Bioinformatics. 2020 Aug 4;21:342. doi: 10.1186/s12859-020-03700-5 (PMC7430822; doi:10.1186/s12859-020-03700-5)
Supplement: Supplementary file 3 — Additional file 3: Supplementary Figure. 1. Runtime of scTyper according to CPU cores up to 20. A plot shows runtimes for cell typing pipeline of scTyper according to the CPU cores (up to 20). The “NTP” cell typing method and inferCNV were applied for the test. [file 12859_2020_3700_MOESM3_ESM.docx]

**Supplementary Figure**


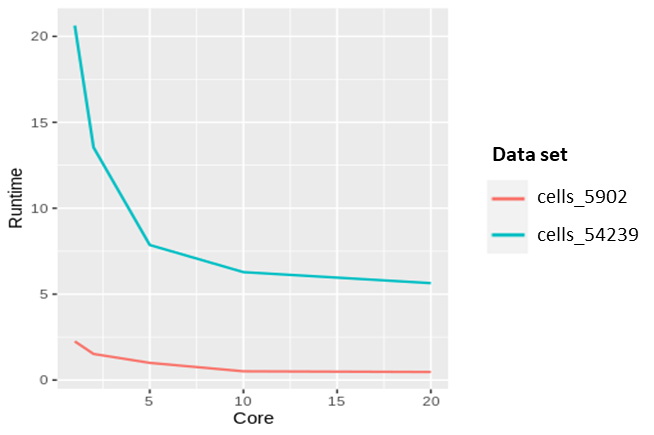


**Figure S1. Runtime of scTyper according to CPU cores up to 20**

A plot indicates runtimes for cell typing with NTP method and infer-CNV mode according to the CPU cores up to 20.
