# Supplementary material for: scTyper: a comprehensive pipeline for the cell typing analysis of single-cell RNA-seq data
Source: BMC Bioinformatics. 2020 Aug 4;21:342. doi: 10.1186/s12859-020-03700-5 (PMC7430822; doi:10.1186/s12859-020-03700-5)
Supplement: Supplementary file 4 — Additional file 4: Supplementary Data. An example report summary document of scTyper. [file 12859_2020_3700_MOESM4_ESM.html]

scTyper Report


# scTyper Report

- Report Summary
  - 1. Parameters used
  - 2. Statistic of the input data
  - 3. Result of pre-processing
  - 4. Result of cellranger - summary metrics
  - 5. Result of cell typing
    - 5-1. Cell typing methods and cell markers used
    - 5-2. Cell type statistics
    - 5-3. Distribution of cell types

---

## Report Summary

\_\_\_\_\_\_\_\_\_\_\_\_\_\_\_\_\_\_\_\_\_\_\_\_\_\_\_\_\_\_\_\_\_\_\_\_\_\_\_\_\_\_\_\_\_\_\_\_\_\_\_\_\_\_\_\_\_\_\_\_\_\_

##### **Project Name** : scTyper

##### **Date** : 2020-06-04 18:24:49

##### **User** : hikim

##### **Processed excuted pipeline** : QC (FALSE), CellRanger (FALSE), Normalization (FALSE), Cell Typing (NTP), inferCNV (TRUE)

##### **Input data type** : Seurat object

##### **Runtime** : 28.8 mins

\_\_\_\_\_\_\_\_\_\_\_\_\_\_\_\_\_\_\_\_\_\_\_\_\_\_\_\_\_\_\_\_\_\_\_\_\_\_\_\_\_\_\_\_\_\_\_\_\_\_\_\_\_\_\_\_\_\_\_\_\_\_\_

scTyper supports a report summarizing for cell typing pipeline. This report provides information of the parameters used to run, samples statistics and visualize assigned cell types using t-SNE plot and heatmap.

---

### 1. Parameters used

- **Global configuration parameters**:

  | Parameters | Values |
  | --- | --- |
  | proj.name | scTyper |
  | wd | /data/Rpackage/scTyper |
  | output.name | /Set.test.public.result/public.NTP.cell.T |
  | pheno.fn | /data/Rpackage/scTyper/data/pheno\_info\_public.csv |
  | qc | FALSE |
  | run.cellranger | FALSE |
  | norm.seurat | FALSE |
  | cell.typing.method | NTP |
  | level | cell |
  | run.inferCNV | TRUE |
  | mc.cores | 10 |
  | report.mode | TRUE |

- **Cell typing parameters**:

  | Parameters | Values |
  | --- | --- |
  | marker | Puram.2017.HNSCC.TME |
  | slot | scale.data |
  | assay | RNA |
  | NTP.g.filter.method | sd |
  | NTP.gene.filter.cutoff | 0.3 |
  | NTP.distance | cosine |
  | NTP.norm.method | none |
  | gene.ref.gtf | /data/pubdata/ngs\_ref/cellranger/refdata-cellranger-GRCh38-1.2.0/genes/genes.gtf |
  | feature.to.test | cell.type |
  | cells.test\_excluded | c(“Epithelial\_cell”, “Unresolved\_cell”) |
  | cells.test\_reference | c(“Fibroblasts”, “T.cells”, “B\_Plasma.cells”, “Macrophages”, “Dendritic.cells”, “Endothelial.cells”, “Myocytes”, “Mast”) |
  | fc.cutoff | 0.05 |
  | malignant.cell.type | Epithelial\_cell |
  | cutoff.gene.cluster | 0.05 |

### 2. Statistic of the input data

##### - **Number of cells:** 5902

##### - **Number of samples:** 24

##### - **Sample statistics**

| sampleID | Number.of.Cells | Average.count.of.nUMIs | Average.number.of.genes |
| --- | --- | --- | --- |
| Cancer\_P0 | 259 | 7433 | 3245 |
| Cancer\_P10 | 89 | 6983 | 3163 |
| Cancer\_P12 | 157 | 6809 | 3355 |
| Cancer\_P13 | 90 | 9422 | 4060 |
| Cancer\_P16 | 596 | 8819 | 3757 |
| Cancer\_P17 | 490 | 11705 | 4900 |
| Cancer\_P18 | 567 | 8876 | 3719 |
| Cancer\_P20 | 345 | 15332 | 6174 |
| Cancer\_P22 | 201 | 11070 | 4494 |
| Cancer\_P23 | 51 | 6626 | 2352 |
| Cancer\_P24 | 130 | 9196 | 3853 |
| Cancer\_P25 | 434 | 10084 | 4139 |
| Cancer\_P26 | 130 | 9683 | 4008 |
| Cancer\_P28 | 370 | 9335 | 3428 |
| Cancer\_P5 | 213 | 11520 | 4604 |
| Cancer\_P6 | 308 | 12237 | 4924 |
| Cancer\_P7 | 7 | 11067 | 5089 |
| Cancer\_P8 | 104 | 7648 | 3504 |
| LymphNode\_P0 | 23 | 7240 | 2787 |
| LymphNode\_P20 | 346 | 13835 | 5503 |
| LymphNode\_P25 | 291 | 9168 | 4067 |
| LymphNode\_P26 | 298 | 11601 | 5158 |
| LymphNode\_P28 | 246 | 10901 | 4449 |
| LymphNode\_P5 | 157 | 12026 | 4842 |

---

### 3. Result of pre-processing

```
[1] "FastQC process is skipped"
```

### 4. Result of cellranger - summary metrics

```
[1] "CellRanger process is skipped"
```

### 5. Result of cell typing

scTyper provides three cell typing methods of the nearest template prediction (NTP), enrichment scores(ES) and average values for cell markers. Cell types are assigned according to the marker gene and cell typing method specified by the user as parameters.

#### 5-1. Cell typing methods and cell markers used

- **Cell typing method**: NTP
- **infer-CNV Malignant cell typing**: Yes
- **Cell marker sets**: Puram.2017.HNSCC.TME

  - **T\_cell**: *CD2*, *CD3D*, *CD3E* and *CD3G*
  - **B\_Plasma\_cell**: *SLAMF7*, *CD79A*, *BLNK* and *FCRL5*
  - **Dendritic\_cell**: *CD40*, *CD80*, *CD83* and *CCR7*
  - **Endothelial\_cell**: *PECAM1*, *VWF* and *ENG*
  - **Epithelial\_cell**: *KRT14*, *KRT17*, *KRT6A*, *KRT5*, *KRT19*, *KRT8*, *KRT16*, *KRT18*, *KRT6B*, *KRT15*, *KRT6C*, *KRTCAP3*, *EPCAM* and *SFN*
  - **Mast\_cell**: *CMA1*, *MS4A2*, *TPSAB1* and *TPSB2*
  - **Macrophage**: *CD14*, *CD163*, *CD68*, *FCGR2A* and *CSF1R*
  - **Myocyte**: *ACTA1*, *ACTN2*, *MYL2* and *MYH2*
  - **Fibroblast**: *FAP*, *PDPN*, *COL1A2*, *DCN*, *COL3A1* and *COL6A1*


---

#### 5-2. Cell type statistics

*In malignant cell typing, a total 2739 malignant cells were assigned by NTP (n= 2332), or inferCNV (n= 2618)*

**Cell type statistics across samples**

|  | Total | Cancer\_P0 | Cancer\_P10 | Cancer\_P12 | Cancer\_P13 | Cancer\_P16 | Cancer\_P17 | Cancer\_P18 | Cancer\_P20 | Cancer\_P22 | Cancer\_P23 | Cancer\_P24 | Cancer\_P25 | Cancer\_P26 | Cancer\_P28 | Cancer\_P5 | Cancer\_P6 | Cancer\_P7 | Cancer\_P8 | LymphNode\_P0 | LymphNode\_P20 | LymphNode\_P25 | LymphNode\_P26 | LymphNode\_P28 | LymphNode\_P5 |
| --- | --- | --- | --- | --- | --- | --- | --- | --- | --- | --- | --- | --- | --- | --- | --- | --- | --- | --- | --- | --- | --- | --- | --- | --- | --- |
| **B\_Plasma\_cell** | 190 | 5 | 44 | 2 | 10 | 12 | 6 | 18 | 0 | 0 | 0 | 0 | 6 | 0 | 0 | 3 | 4 | 0 | 13 | 12 | 0 | 27 | 3 | 22 | 3 |
| **Dendritic\_cell** | 221 | 17 | 4 | 12 | 35 | 24 | 8 | 30 | 0 | 5 | 0 | 1 | 30 | 2 | 8 | 5 | 1 | 0 | 10 | 1 | 0 | 20 | 4 | 2 | 2 |
| **Endothelial\_cell** | 249 | 2 | 7 | 0 | 2 | 36 | 18 | 18 | 2 | 1 | 0 | 2 | 5 | 3 | 17 | 8 | 42 | 0 | 26 | 1 | 0 | 9 | 10 | 12 | 28 |
| **Fibroblast** | 638 | 7 | 23 | 0 | 2 | 57 | 38 | 29 | 7 | 27 | 0 | 57 | 47 | 32 | 106 | 15 | 39 | 1 | 19 | 4 | 0 | 30 | 38 | 52 | 8 |
| **Macrophage** | 125 | 4 | 1 | 1 | 3 | 13 | 2 | 23 | 1 | 0 | 0 | 4 | 5 | 2 | 9 | 41 | 3 | 0 | 3 | 0 | 0 | 4 | 1 | 4 | 1 |
| **Malignant\_cell** | 2739 | 20 | 5 | 12 | 16 | 102 | 357 | 214 | 329 | 123 | 0 | 30 | 191 | 63 | 78 | 108 | 211 | 6 | 12 | 0 | 343 | 91 | 217 | 112 | 99 |
| **Mast\_cell** | 125 | 6 | 1 | 0 | 4 | 25 | 7 | 12 | 0 | 22 | 0 | 20 | 11 | 1 | 0 | 4 | 1 | 0 | 3 | 1 | 0 | 3 | 0 | 2 | 2 |
| **Myocyte** | 51 | 2 | 0 | 0 | 0 | 4 | 0 | 2 | 0 | 0 | 0 | 0 | 15 | 1 | 16 | 1 | 0 | 0 | 0 | 0 | 0 | 5 | 1 | 4 | 0 |
| **T\_cell** | 1008 | 184 | 0 | 121 | 10 | 269 | 45 | 161 | 0 | 0 | 50 | 0 | 66 | 3 | 0 | 23 | 0 | 0 | 18 | 1 | 0 | 56 | 1 | 0 | 0 |
| **Unresolved\_cell** | 556 | 12 | 4 | 9 | 8 | 54 | 9 | 60 | 6 | 23 | 1 | 16 | 58 | 23 | 136 | 5 | 7 | 0 | 0 | 3 | 3 | 46 | 23 | 36 | 14 |

---

#### 5-3. Distribution of cell types

**Distribution of inferred cell types**

**Distibution of cell types across samples**

**t-SNE plots**

- **Inferred cell types by NTP**

- **Malignant cells by inferCNV**

- **CNV score by inferCNV**

- **Seurat clusters**

- **Samples**

- **Cell markers heatmap**

A heatmap shows the cell typing result and the gene expression levels of cell marker gene sets from Puram.2017.HNSCC.TME. For each method, the assigned cell types are indicated by color bars.
